# Dimethyl 2,2′-[(4-oxo-2-phenyl-4*H*-chromene-5,7-di­yl)di­oxy]diacetate: a less densely packed polymorph

**DOI:** 10.1107/S1600536809001019

**Published:** 2009-01-14

**Authors:** Angannan Nallasivam, Munirathinam Nethaji, Nagarajan Vembu, Buckle Jaswant, Nagarajan Sulochana

**Affiliations:** a Department of Chemistry, National Institute of Technology, Tiruchirappalli 620 015, India; b Department of Inorganic and Physical Chemistry, Indian Institute of Science, Bangalore 560 012, India; c Department of Chemistry, Urumu Dhanalakshmi College, Tiruchirappalli 620 019, India; d Department of Chemistry, Government Arts College, Karur 639 005, India

## Abstract

The title mol­ecule, C_21_H_18_O_8_, crystallizes in two crystal polymorphs, see also Nallasivam, Nethaji, Vembu & Jaswant [*Acta Cryst*. (2009), E**65**, o312–o313]. The main difference between the two polymorphs is in the conformation of the oxomethyl­acetate groups with regard to the almost planar [total puckering amplitude 0.047 (2) Å] chromene ring. In the title compound, the best planes of the oxomethyl­acetate groups through the non-H atoms are almost perpendicular to the chromene ring [making dihedral angles of 89.61 (6) and 80.59 (5)°], while in the second polymorph the mol­ecules are close to planar. Both crystal structures are stabilized by C—H⋯O.

## Related literature

For the second polymorph, see: Nallasivam *et al.* (2009 ▶). For the biological and pharmacological properties of benzopyrans and their derivatives, see: Brooks (1998 ▶); Hatakeyama *et al.* (1988 ▶); Hyana & Saimoto (1987 ▶); Tang *et al.* (2007 ▶). For the importance of 4*H*-chromenes, see Liu *et al.* (2007 ▶); Wang, Fang *et al.* (2003 ▶); Wand, Zheng *et al.* (2003 ▶).For hydrogen-bond motifs, see: Bernstein *et al.* (1995 ▶); Desiraju & Steiner (1999 ▶); Etter (1990 ▶).
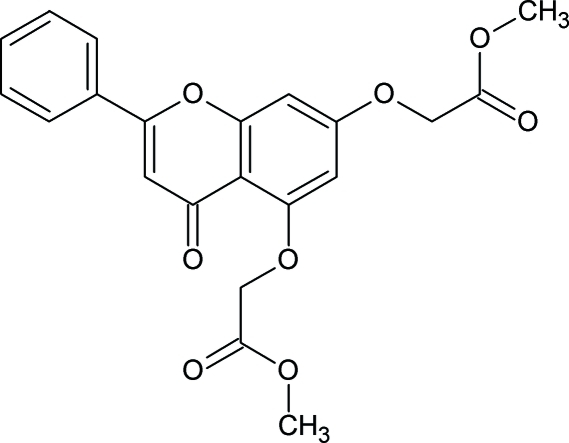


## Experimental

### 

#### Crystal data

C_21_H_18_O_8_
*M* *_r_* = 398.35
Triclinic, 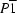

*a* = 9.4024 (16) Å
*b* = 9.8506 (17) Å
*c* = 11.1570 (18) Å
α = 67.817 (3)°
β = 80.300 (3)°
γ = 89.683 (3)°
*V* = 941.3 (3) Å^3^
*Z* = 2
Mo *K*α radiation
μ = 0.11 mm^−1^
*T* = 293 (2) K
0.42 × 0.35 × 0.29 mm

#### Data collection

Bruker SMART APEX CCD diffractometer
Absorption correction: multi-scan (*SADABS*; Sheldrick, 1998 ▶) *T*_min_ = 0.955, *T*_max_ = 0.969
8351 measured reflections
4316 independent reflections
2424 reflections with *I* > 2σ(*I*)
*R*_int_ = 0.026

#### Refinement

*R*[*F* ^2^ > 2σ(*F* ^2^)] = 0.055
*wR*(*F* ^2^) = 0.140
*S* = 0.98
4316 reflections
264 parameters
H-atom parameters constrained
Δρ_max_ = 0.17 e Å^−3^
Δρ_min_ = −0.17 e Å^−3^

### 

Data collection: *SMART* (Bruker, 2007 ▶); cell refinement: *SAINT* (Bruker, 2007 ▶); data reduction: *SAINT*; program(s) used to solve structure: *SHELXS97* (Sheldrick, 2008 ▶); program(s) used to refine structure: *SHELXL97* (Sheldrick, 2008 ▶); molecular graphics: *PLATON* (Spek, 2003 ▶); software used to prepare material for publication: *SHELXL97*.

## Supplementary Material

Crystal structure: contains datablocks I, global. DOI: 10.1107/S1600536809001019/fb2124sup1.cif

Structure factors: contains datablocks I. DOI: 10.1107/S1600536809001019/fb2124Isup2.hkl

Additional supplementary materials:  crystallographic information; 3D view; checkCIF report

## Figures and Tables

**Table 1:** Hydrogen-bond geometry (Å, °)

*D*—H⋯*A*	*D*—H	H⋯*A*	*D*⋯*A*	*D*—H⋯*A*
C19—H19*A*⋯O17^i^	0.97	2.54	3.214 (3)	127
C19—H19*A*⋯O18^i^	0.97	2.58	3.366 (2)	138

Symmetry codes: (i) 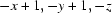.

**Fig. 1.**
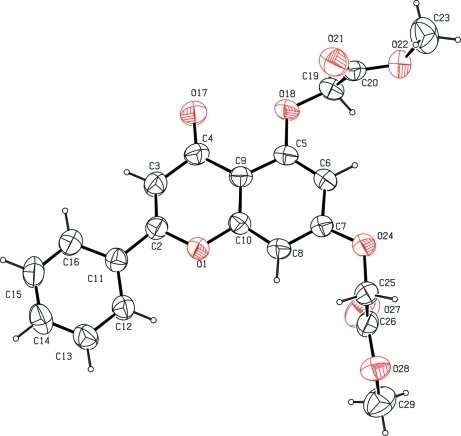
The title molecule showing the displacement ellipsoids depicted at the 50% probability level for all non-H atoms. The hydrogen atoms are drawn as spheres of arbitrary radius.

## supplementary crystallographic information

### Comment

Chromenes (benzopyrans) and their derivatives have numerous biological and pharmacological properties (Tang *et al.*, 2007) such as antisterility (Brooks, 1998) and anticancer activity (Hyana & Saimoto, 1987). In addition, polyfunctional chromene units are present in numerous natural products (Hatakeyama *et al.*, 1988). 4*H*-chromenes are important synthons for some natural products (Liu *et al.*, 2007). As a part of our structural investigations on 4*H*-chromene derivatives and compounds containing the benzopyran fragment, the single-crystal X-ray diffraction study on the title compound was carried out.

The chromene ring is almost planar. This planarity is common for the related chromene derivatives (Wei *et al.*, 2003; Wang *et al.*, 2003). The total puckering amplitude of the chromene ring in the title structure is 0.047 (2) Å. The interplanar angle between the chromene ring and the 2-phenyl ring is 17.20 (7)° thereby indicating the distorted coplanar arrangement (Fig. 1). This angle is substantially larger than in the other polymorph (Nallasivam *et al.*, 2009) that is only 2.90 (6)°. In the title structure, both oxomethylacetate groups are almost perpendicular to the plane of the chromene ring; the planes through their non-hydrogen atoms contain the angles with the plane through the non-hydrogen chromene-ring atoms equal to 89.61 (6) and 80.59 (5)° for the groups attached at C5 and C7, respectively. This is the main difference to the second polymorph (Nallasivam *et al.*, 2009) where the respective angles are 6.60 (64), 22 (4) and 12.45 (5)° for the chains A and B attached to C5 and to the chain attached to C7.

It should be noted that the title compound is considerably less densely packed than the second polymorph (the respective unit cell volumes are 941.3 (3) (the title structure) and 915.5 (3) Å^3^ (Nallasivam *et al.*, 2009). The reason plausibly consists in the different shapes of the molecules in both polymorphs; a more planar shape of the second polymorph enables a denser packing of the molecules in spite of a partial disorder of the oxomethylacetate group attached to C5.

The weak interactions are of a similar character in both polymorphs: C—H···O, C—H···π electron and π-π electron interactions. However, the number of weak interactions in the title structure is lower than in the second polymorph (Nallasivam *et al.*, 2009).

The weak C–H···O and C–H···π interactions are given in Tab. 1; Desiraju & Steiner, 1999). The C19—H19A···O17^i^ and C19—H19A···O18^i^ interactions constitute a pair of bifurcated donor bonds generating a ring of graph set *R*^2^_1_(6) (Bernstein *et al.*, 1995; Etter, 1990). There is also a face to face interaction between two symmetry related (1-*x*, -*y*, 1-*z*) (O1\C2\C3\C4\C9\C10) rings with mutual distance between the centroids being 3.633 (1) Å.

### Experimental

The title compound was prepared from the more densely packed polymorph (Nallasivam *et al.*, 2009). The crude product of the above polymorph (1.26 mmol, 0.5 g) was dissolved in dichloromethane (10 ml). The content was cooled to 283–288 K and AlCl_3_ (1.50 mmol, 0.2 g) was added. Acetyl chloride (21.21 mmol, 1.5 ml) was added dropwise over a period of 15–20 min. The reaction mixture was maintained for 10–12 hrs at 283–288 K, quenched with HCl/ice mixture, extracted with dichloromethane and purified by column chromatography, using 20 ml of 1:1 (volume) mixture of ethyl acetate and n-hexane. Diffraction quality prism shaped crystals of the title compound, the less densely packed polymorph, with average size about 0.35 mm along its long edge were obtained. Yield: 80%

### Refinement

All the H-atoms were observed in the difference electron density map. However, they were situated into idealized positions with C–H = 0.93, 0.97 and 0.96Å for aryl, methylene and methyl H, respectively, and constrained to ride on their parent atoms with *U*_iso_(H)=1.2*U*_eq_(C) for the aryl and methylene H and *U*_iso_(H)=1.5*U*_eq_(C) for the methyl H.

### Crystal data

**Table:**

C_21_H_18_O_8_	*Z* = 2
*M**_r_* = 398.35	*F*(000) = 416
Triclinic, *P*1	*D*_x_ = 1.406 Mg m^−^^3^
Hall symbol: -P 1	Melting point = 418–420 K
*a* = 9.4024 (16) Å	Mo *K*α radiation, λ = 0.71073 Å
*b* = 9.8506 (17) Å	Cell parameters from 576 reflections
*c* = 11.1570 (18) Å	θ = 2.0–27.0°
α = 67.817 (3)°	µ = 0.11 mm^−^^1^
β = 80.300 (3)°	*T* = 293 K
γ = 89.683 (3)°	Prism, colourless
*V* = 941.3 (3) Å^3^	0.42 × 0.35 × 0.29 mm

### Data collection

**Table:**

Bruker SMART APEX CCD diffractometer	4316 independent reflections
Radiation source: fine-focus sealed tube	2424 reflections with *I* > 2σ(*I*)
graphite	*R*_int_ = 0.026
Detector resolution: 0.3 pixels mm^-1^	θ_max_ = 28.0°, θ_min_ = 2.0°
ω scans	*h* = −12→12
Absorption correction: multi-scan (*SADABS*; Sheldrick, 1998)	*k* = −12→12
*T*_min_ = 0.955, *T*_max_ = 0.969	*l* = −14→12
8351 measured reflections	

### Refinement

**Table:**

Refinement on *F*^2^	Primary atom site location: structure-invariant direct methods
Least-squares matrix: full	Secondary atom site location: difference Fourier map
*R*[*F*^2^ > 2σ(*F*^2^)] = 0.055	Hydrogen site location: difference Fourier map
*wR*(*F*^2^) = 0.140	H-atom parameters constrained
*S* = 0.98	*w* = 1/[σ^2^(*F*_o_^2^) + (0.0653*P*)^2^] where *P* = (*F*_o_^2^ + 2*F*_c_^2^)/3
4316 reflections	(Δ/σ)_max_ < 0.001
264 parameters	Δρ_max_ = 0.17 e Å^−^^3^
0 restraints	Δρ_min_ = −0.17 e Å^−^^3^
72 constraints	

### Special details

**Table:**

Geometry. All e.s.d.'s (except the e.s.d. in the dihedral angle between two l.s. planes) are estimated using the full covariance matrix. The cell e.s.d.'s are taken into account individually in the estimation of e.s.d.'s in distances, angles and torsion angles; correlations between e.s.d.'s in cell parameters are only used when they are defined by crystal symmetry. An approximate (isotropic) treatment of cell e.s.d.'s is used for estimating e.s.d.'s involving l.s. planes.
Refinement. Refinement of *F*^2^ against ALL reflections. The weighted *R*-factor *wR* and goodness of fit *S* are based on *F*^2^, conventional *R*-factors *R* are based on *F*, with *F* set to zero for negative *F*^2^. The threshold expression of *F*^2^ > σ(*F*^2^) is used only for calculating *R*-factors(gt) *etc*. and is not relevant to the choice of reflections for refinement. *R*-factors based on *F*^2^ are statistically about twice as large as those based on *F*, and *R*- factors based on ALL data will be even larger.

### Fractional atomic coordinates and isotropic or equivalent isotropic displacement parameters (Å^2^)

**Table:**

	*x*	*y*	*z*	*U*_iso_*/*U*_eq_	
O1	0.40612 (14)	0.11111 (15)	0.61070 (13)	0.0480 (4)	
C2	0.3081 (2)	0.0338 (2)	0.5793 (2)	0.0442 (5)	
C3	0.2882 (2)	0.0715 (2)	0.4561 (2)	0.0527 (6)	
H3	0.2234	0.0133	0.4387	0.063*	
C4	0.3620 (2)	0.1982 (3)	0.3475 (2)	0.0537 (6)	
C5	0.5576 (2)	0.4028 (2)	0.29261 (19)	0.0449 (5)	
C6	0.6546 (2)	0.4693 (2)	0.3356 (2)	0.0492 (5)	
H6	0.7123	0.5510	0.2758	0.059*	
C7	0.6676 (2)	0.4163 (2)	0.4671 (2)	0.0465 (5)	
C8	0.5847 (2)	0.2951 (2)	0.5581 (2)	0.0474 (5)	
H8	0.5942	0.2582	0.6462	0.057*	
C9	0.4678 (2)	0.2784 (2)	0.38264 (19)	0.0426 (5)	
C10	0.4865 (2)	0.2302 (2)	0.51311 (19)	0.0424 (5)	
C11	0.2310 (2)	−0.0864 (2)	0.6973 (2)	0.0438 (5)	
C12	0.2836 (2)	−0.1350 (2)	0.8139 (2)	0.0505 (5)	
H12	0.3721	−0.0956	0.8166	0.061*	
C13	0.2065 (2)	−0.2414 (2)	0.9265 (2)	0.0571 (6)	
H13	0.2432	−0.2733	1.0042	0.069*	
C14	0.0759 (3)	−0.2999 (3)	0.9234 (2)	0.0643 (7)	
H14	0.0226	−0.3694	0.9997	0.077*	
C15	0.0242 (3)	−0.2560 (3)	0.8081 (3)	0.0711 (7)	
H15	−0.0632	−0.2979	0.8058	0.085*	
C16	0.1004 (2)	−0.1503 (3)	0.6951 (2)	0.0625 (6)	
H16	0.0643	−0.1216	0.6171	0.075*	
O17	0.33591 (19)	0.2322 (2)	0.23630 (16)	0.0923 (6)	
O18	0.54047 (15)	0.44833 (16)	0.16484 (13)	0.0563 (4)	
C19	0.6399 (2)	0.5593 (2)	0.06901 (19)	0.0516 (5)	
H19A	0.6412	0.5540	−0.0162	0.062*	
H19B	0.7360	0.5413	0.0900	0.062*	
C20	0.6040 (2)	0.7112 (2)	0.06058 (19)	0.0472 (5)	
O21	0.48863 (17)	0.74539 (18)	0.09905 (16)	0.0719 (5)	
O22	0.71948 (15)	0.80238 (16)	−0.00131 (15)	0.0588 (4)	
C23	0.6961 (3)	0.9562 (3)	−0.0494 (3)	0.0925 (9)	
H23A	0.6518	0.9818	−0.1259	0.139*	
H23B	0.7870	1.0117	−0.0721	0.139*	
H23C	0.6337	0.9781	0.0176	0.139*	
O24	0.76487 (16)	0.49744 (16)	0.49501 (14)	0.0630 (4)	
C25	0.8002 (2)	0.4478 (2)	0.6227 (2)	0.0540 (6)	
H25A	0.7126	0.4118	0.6880	0.065*	
H25B	0.8438	0.5292	0.6354	0.065*	
C26	0.9035 (2)	0.3269 (3)	0.6417 (2)	0.0522 (6)	
O27	0.94183 (18)	0.2686 (2)	0.56667 (18)	0.0864 (6)	
O28	0.94756 (16)	0.29423 (18)	0.75606 (15)	0.0627 (4)	
C29	1.0535 (3)	0.1846 (3)	0.7853 (3)	0.0833 (8)	
H29A	1.0141	0.0941	0.7869	0.125*	
H29B	1.0775	0.1691	0.8696	0.125*	
H29C	1.1389	0.2179	0.7189	0.125*	

### Atomic displacement parameters (Å^2^)

**Table:**

	*U*^11^	*U*^22^	*U*^33^	*U*^12^	*U*^13^	*U*^23^
O1	0.0492 (8)	0.0528 (9)	0.0363 (8)	−0.0084 (7)	−0.0069 (6)	−0.0110 (7)
C2	0.0403 (11)	0.0490 (13)	0.0444 (13)	0.0008 (9)	−0.0075 (9)	−0.0192 (11)
C3	0.0504 (12)	0.0624 (15)	0.0464 (14)	−0.0076 (11)	−0.0092 (10)	−0.0217 (12)
C4	0.0526 (12)	0.0672 (15)	0.0401 (13)	−0.0024 (11)	−0.0141 (10)	−0.0167 (12)
C5	0.0510 (12)	0.0457 (12)	0.0345 (12)	0.0042 (10)	−0.0118 (9)	−0.0099 (10)
C6	0.0558 (12)	0.0445 (12)	0.0395 (12)	−0.0049 (10)	−0.0098 (10)	−0.0066 (10)
C7	0.0506 (12)	0.0455 (13)	0.0419 (13)	−0.0025 (10)	−0.0141 (10)	−0.0126 (10)
C8	0.0552 (12)	0.0493 (13)	0.0350 (12)	−0.0010 (10)	−0.0134 (10)	−0.0109 (10)
C9	0.0438 (11)	0.0453 (12)	0.0369 (12)	0.0033 (9)	−0.0097 (9)	−0.0128 (10)
C10	0.0419 (11)	0.0437 (12)	0.0390 (12)	0.0014 (9)	−0.0064 (9)	−0.0131 (10)
C11	0.0408 (11)	0.0475 (13)	0.0435 (13)	0.0011 (9)	−0.0028 (9)	−0.0197 (10)
C12	0.0480 (12)	0.0552 (14)	0.0453 (13)	−0.0071 (10)	−0.0045 (10)	−0.0173 (11)
C13	0.0656 (14)	0.0572 (15)	0.0430 (13)	−0.0072 (12)	−0.0034 (11)	−0.0154 (11)
C14	0.0662 (15)	0.0590 (16)	0.0587 (16)	−0.0149 (12)	0.0072 (12)	−0.0201 (13)
C15	0.0577 (14)	0.0760 (18)	0.0725 (19)	−0.0235 (13)	−0.0037 (13)	−0.0235 (15)
C16	0.0535 (13)	0.0744 (17)	0.0553 (15)	−0.0121 (12)	−0.0116 (11)	−0.0192 (13)
O17	0.1010 (13)	0.1168 (16)	0.0438 (10)	−0.0444 (11)	−0.0271 (9)	−0.0072 (10)
O18	0.0704 (10)	0.0583 (10)	0.0319 (8)	−0.0116 (8)	−0.0143 (7)	−0.0058 (7)
C19	0.0572 (13)	0.0548 (14)	0.0345 (12)	−0.0026 (11)	−0.0052 (10)	−0.0091 (10)
C20	0.0446 (12)	0.0586 (14)	0.0347 (12)	0.0011 (11)	−0.0120 (9)	−0.0118 (10)
O21	0.0556 (10)	0.0754 (12)	0.0693 (12)	0.0106 (9)	−0.0034 (8)	−0.0140 (9)
O22	0.0544 (9)	0.0552 (10)	0.0648 (10)	−0.0084 (8)	−0.0096 (8)	−0.0209 (8)
C23	0.102 (2)	0.0577 (18)	0.114 (3)	−0.0123 (15)	−0.0184 (18)	−0.0293 (17)
O24	0.0775 (10)	0.0589 (10)	0.0459 (9)	−0.0207 (8)	−0.0219 (8)	−0.0078 (8)
C25	0.0633 (13)	0.0581 (14)	0.0429 (13)	−0.0102 (11)	−0.0143 (10)	−0.0195 (11)
C26	0.0450 (12)	0.0653 (15)	0.0486 (14)	−0.0105 (11)	−0.0063 (10)	−0.0250 (12)
O27	0.0782 (12)	0.1268 (16)	0.0877 (14)	0.0158 (11)	−0.0217 (10)	−0.0756 (13)
O28	0.0654 (10)	0.0762 (11)	0.0526 (10)	0.0131 (8)	−0.0187 (8)	−0.0282 (9)
C29	0.0669 (16)	0.091 (2)	0.100 (2)	0.0190 (15)	−0.0314 (15)	−0.0389 (18)

### Geometric parameters (Å, °)

**Table:**

O1—C2	1.368 (2)	C14—H14	0.9300
O1—C10	1.379 (2)	C15—C16	1.378 (3)
C2—C3	1.327 (3)	C15—H15	0.9300
C2—C11	1.474 (3)	C16—H16	0.9300
C3—C4	1.446 (3)	O18—C19	1.416 (2)
C3—H3	0.9300	C19—C20	1.503 (3)
C4—O17	1.225 (2)	C19—H19A	0.9700
C4—C9	1.463 (3)	C19—H19B	0.9700
C5—O18	1.361 (2)	C20—O21	1.193 (2)
C5—C6	1.372 (3)	C20—O22	1.324 (2)
C5—C9	1.421 (3)	O22—C23	1.433 (3)
C6—C7	1.386 (3)	C23—H23A	0.9600
C6—H6	0.9300	C23—H23B	0.9600
C7—O24	1.365 (2)	C23—H23C	0.9600
C7—C8	1.376 (3)	O24—C25	1.417 (2)
C8—C10	1.384 (3)	C25—C26	1.507 (3)
C8—H8	0.9300	C25—H25A	0.9700
C9—C10	1.392 (3)	C25—H25B	0.9700
C11—C12	1.382 (3)	C26—O27	1.192 (2)
C11—C16	1.391 (3)	C26—O28	1.330 (2)
C12—C13	1.382 (3)	O28—C29	1.448 (3)
C12—H12	0.9300	C29—H29A	0.9600
C13—C14	1.370 (3)	C29—H29B	0.9600
C13—H13	0.9300	C29—H29C	0.9600
C14—C15	1.366 (3)		
			
C2—O1—C10	119.73 (16)	C14—C15—C16	120.6 (2)
C3—C2—O1	121.22 (18)	C14—C15—H15	119.7
C3—C2—C11	127.40 (19)	C16—C15—H15	119.7
O1—C2—C11	111.35 (17)	C15—C16—C11	120.3 (2)
C2—C3—C4	123.5 (2)	C15—C16—H16	119.9
C2—C3—H3	118.3	C11—C16—H16	119.9
C4—C3—H3	118.3	C5—O18—C19	117.84 (16)
O17—C4—C3	121.2 (2)	O18—C19—C20	112.95 (17)
O17—C4—C9	124.2 (2)	O18—C19—H19A	109.0
C3—C4—C9	114.51 (18)	C20—C19—H19A	109.0
O18—C5—C6	123.80 (18)	O18—C19—H19B	109.0
O18—C5—C9	115.77 (18)	C20—C19—H19B	109.0
C6—C5—C9	120.43 (18)	H19A—C19—H19B	107.8
C5—C6—C7	120.84 (19)	O21—C20—O22	125.3 (2)
C5—C6—H6	119.6	O21—C20—C19	125.6 (2)
C7—C6—H6	119.6	O22—C20—C19	109.07 (18)
O24—C7—C8	125.08 (18)	C20—O22—C23	116.67 (18)
O24—C7—C6	113.78 (18)	O22—C23—H23A	109.5
C8—C7—C6	121.11 (19)	O22—C23—H23B	109.5
C7—C8—C10	117.24 (19)	H23A—C23—H23B	109.5
C7—C8—H8	121.4	O22—C23—H23C	109.5
C10—C8—H8	121.4	H23A—C23—H23C	109.5
C10—C9—C5	115.94 (18)	H23B—C23—H23C	109.5
C10—C9—C4	118.98 (18)	C7—O24—C25	119.88 (16)
C5—C9—C4	125.06 (18)	O24—C25—C26	111.09 (18)
O1—C10—C8	113.59 (17)	O24—C25—H25A	109.4
O1—C10—C9	121.98 (18)	C26—C25—H25A	109.4
C8—C10—C9	124.43 (19)	O24—C25—H25B	109.4
C12—C11—C16	118.3 (2)	C26—C25—H25B	109.4
C12—C11—C2	121.07 (18)	H25A—C25—H25B	108.0
C16—C11—C2	120.61 (19)	O27—C26—O28	124.2 (2)
C13—C12—C11	120.9 (2)	O27—C26—C25	126.1 (2)
C13—C12—H12	119.5	O28—C26—C25	109.75 (18)
C11—C12—H12	119.5	C26—O28—C29	116.20 (18)
C14—C13—C12	119.9 (2)	O28—C29—H29A	109.5
C14—C13—H13	120.0	O28—C29—H29B	109.5
C12—C13—H13	120.0	H29A—C29—H29B	109.5
C15—C14—C13	119.9 (2)	O28—C29—H29C	109.5
C15—C14—H14	120.0	H29A—C29—H29C	109.5
C13—C14—H14	120.0	H29B—C29—H29C	109.5
			
C10—O1—C2—C3	−0.2 (3)	C4—C9—C10—C8	178.82 (18)
C10—O1—C2—C11	−178.52 (15)	C3—C2—C11—C12	165.1 (2)
O1—C2—C3—C4	−2.6 (3)	O1—C2—C11—C12	−16.8 (3)
C11—C2—C3—C4	175.34 (19)	C3—C2—C11—C16	−17.2 (3)
C2—C3—C4—O17	−177.0 (2)	O1—C2—C11—C16	160.97 (18)
C2—C3—C4—C9	3.3 (3)	C16—C11—C12—C13	−1.9 (3)
O18—C5—C6—C7	−179.33 (18)	C2—C11—C12—C13	175.95 (19)
C9—C5—C6—C7	0.4 (3)	C11—C12—C13—C14	−0.1 (3)
C5—C6—C7—O24	−177.80 (18)	C12—C13—C14—C15	1.9 (3)
C5—C6—C7—C8	0.5 (3)	C13—C14—C15—C16	−1.7 (4)
O24—C7—C8—C10	177.02 (18)	C14—C15—C16—C11	−0.2 (4)
C6—C7—C8—C10	−1.0 (3)	C12—C11—C16—C15	2.0 (3)
O18—C5—C9—C10	179.10 (16)	C2—C11—C16—C15	−175.8 (2)
C6—C5—C9—C10	−0.7 (3)	C6—C5—O18—C19	7.8 (3)
O18—C5—C9—C4	0.5 (3)	C9—C5—O18—C19	−171.99 (17)
C6—C5—C9—C4	−179.34 (19)	C5—O18—C19—C20	−81.3 (2)
O17—C4—C9—C10	179.1 (2)	O18—C19—C20—O21	−20.0 (3)
C3—C4—C9—C10	−1.2 (3)	O18—C19—C20—O22	162.55 (15)
O17—C4—C9—C5	−2.3 (3)	O21—C20—O22—C23	−13.6 (3)
C3—C4—C9—C5	177.45 (19)	C19—C20—O22—C23	163.86 (19)
C2—O1—C10—C8	−177.96 (16)	C8—C7—O24—C25	8.3 (3)
C2—O1—C10—C9	2.3 (3)	C6—C7—O24—C25	−173.57 (18)
C7—C8—C10—O1	−178.97 (17)	C7—O24—C25—C26	77.5 (2)
C7—C8—C10—C9	0.8 (3)	O24—C25—C26—O27	−6.8 (3)
C5—C9—C10—O1	179.79 (16)	O24—C25—C26—O28	172.89 (16)
C4—C9—C10—O1	−1.5 (3)	O27—C26—O28—C29	2.7 (3)
C5—C9—C10—C8	0.1 (3)	C25—C26—O28—C29	−176.98 (18)

### Hydrogen-bond geometry (Å, °)

**Table:**

*D*—H···*A*	*D*—H	H···*A*	*D*···*A*	*D*—H···*A*
C19—H19A···O17^i^	0.97	2.54	3.214 (3)	127
C19—H19A···O18^i^	0.97	2.58	3.366 (2)	138
C29—H29A···Cg3^ii^	0.96	3.06	3.765	131

Symmetry codes: (i) −*x*+1, −*y*+1, −*z*; (ii) *x*+1, *y*, *z*.
